# The serum levels of IL-8, IL-10, and TNF-a, cardiac troponin I, creatine kinase-MB in patients with rheumatic heart disease received dexmedetomidine

**DOI:** 10.5937/jomb0-56030

**Published:** 2025-08-21

**Authors:** Dongmei Yang, Jian Zhang, Caiyun Zhang

**Affiliations:** 1 West China Hospital of Sichuan University, Department of Clinical Pharmacy (Pharmacy), Chengdu, Sichuan Province, China; 2 West China Hospital of Sichuan University, Department of Cardiovascular Surgery, Chengdu, Sichuan Province, China

**Keywords:** dexmedetomidine (DEX), rheumatic heart disease (RHD), valve replacement surgery, cardiopulmonary bypass (CPB), interleukin-8 (IL-8), interleukin-10 (IL-10), tumour necrosis factor-alpha (TNF-a), cardiac troponin I (cTnI), creatine kinase-MB (CK-MB), myocardial injury, inflammatory markers, anaesthetic dosage, hemodynamic stability, deksmedetomidin (D EX), reumatska bolest srca (RHD), operacija zamene valvula, veštački krvotok (CPB), interleukin-8 (IL-8), interleukin-10 (IL-10), faktor nekroze tumora-alfa (TNF-a), srcani troponin I (cTnI), kreatin kinaza-MB (CK-MB), oštećenje miokarda, inflamatorni markeri, doza anestetika, hemodinamička stabilnost

## Abstract

**Background:**

This study aimed to evaluate the effects of dexmedetomidine (DEX) in patients undergoing valve replacement surgery for rheumatic heart disease (RHD), focusing on its impact on serum levels of inflammatory markers (IL-8, IL-10, TNF-a) and myocardial injury markers (Cardiac Troponin I [cTnI], Creatine Kinase-MB [CK-MB]).

**Methods:**

A total of 140 patients undergoing cardiopulmonary bypass (CPB) valve replacement surgery at West China Hospital, Sichuan University, between January 2022 and December 2024 were randomly assigned to two groups: the observation group (DEX) and the control group (normal saline). Key perioperative parameters were analysed, including anaesthetic dosage, myocardial injury markers, immune function (CD4+, CD8+, and CD4+/CD8+ ratio), inflammatory factors, and adverse reactions.

**Results:**

The observation group required significantly lower anaesthetic dosages than the control group. Additionally, the observation group exhibited higher heart rate (HR) at T5 and higher mean arterial pressure (MAP) at T2, T3, and T5 (P< 0.05). Myocardial injury markers (cTnI and CK-MB) were significantly lower in the observation group (P< 0.05). While preoperative cellular immune function (CD4+, CD8+, and CD4+/CD8+) was similar between both groups, postoperative measurements showed significantly higher CD4+ and CD4+/CD8+ ratios, and lower CD8+ in the observation group (P < 0.05). Regarding inflammatory markers, IL-8 and TNF-a levels were significantly lower, while IL-10 was higher in the observation group postoperatively (P < 0 .0 5). There were no significant differences in adverse reactions between the two groups (P > 0 .0 5).

**Conclusions:**

Dexmedetomidine (DEX) reduces anaesthetic usage, supports hemodynamic stability, mitigates myocardial injury, and lowers postoperative inflammatory responses in patients undergoing RHD valve replacement surgery.

## Abstract (sr)

**Uvod:**

Ova studija imala je za cilj da proceni efekte deksmedetomidina (DEX) kod pacijenata koji su podvrgnuti operaciji zamene valvula zbog reumatske bolesti srca (RHD), sa fokusom na njegov uticaj na serumske nivoe inflamatornih markera (IL-8, IL-10, TNF-a) i markera oštećenja miokarda (srcani troponin I [cTnI], kreatin kinaza-MB [CK-MB]).

**Metode:**

Ukupno 140 pacijenata podvrgnutih operaciji zamene srcanih valvula uz upotrebu veštačkog krvotoka (CPB) u Bolnici Zapadne Kine, Univerzitet Sicuan, između januara 2022. i decembra 2024. godine, nasumicno su raspoređeni u dve grupe: posmatranu grupu (DEX) i kontrolnu grupu (fiziološki rastvor). Analizirani su kljucni perioperativni parametri, uključujući dozu anestezije, markere oštećenja miokarda, imunsku funkciju (CD4+, CD8+ i odnos CD4+/CD8+), inflamatorne faktore i neželjene reakcije.

**Rezultati:**

Posmatrana grupa je zahtevala znacajno niže doze anestetika u poređenju sa kontrolnom grupom. Takođe, u istoj grupi su zabeleženi viši srcani ritam (HR) u trenutku T5 i viši srednji arterijski pritisak (MAP) u trenucima T2, T3 i T5 (P< 0,05). Markeri oštećenja miokarda (cTnI i CK-MB) bili su znacajno niži u posmatranoj grupi (P< 0,05). Dok su preoperativne vrednosti ćelijske imunološke funkcije (C d 4+, C d 8+ i CD4+/CD8+) bile slicne u obe grupe, postoperativna merenja su pokazala znacajno više vrednosti CD 4+ i odnosa CD 4+ /C D 8+, dok je CD8+ bio niži u posmatranoj grupi (P< 0,05). što se tice inflamatornih markera, postoperativni nivoi IL-8 i TNF-a bili su značajno niži, dok je IL-10 bio viši u posmatranoj grupi (P < 0 ,0 5 ). Nije bilo značajnih razlika u učestalosti neželjenih reakcija između grupa (P> 0,05).

**Zaključak:**

Deksmedetomidin (DEX) smanjuje potrebu za anesteticima, podržava hemodinamsku stabilnost, ublažava oštećenje miokarda i smanjuje postoperativni inflamatorni odgovor kod pacijenata podvrgnutih operaciji zamene srcanih valvula zbog RHD.

## Introduction

Rheumatic heart disease (RHD) is a chronic condition that affects millions of people worldwide, particularly in areas with limited healthcare resources. It typically results from untreated rheumatic fever, which damages the heart valves, especially the mitral and aortic valves. Over time, this damage can lead to valve stenosis and heart failure, making it harder for the heart to pump blood effectively [1] [2]. For patients with severe valve disease, heart valve replacement surgery is often the only option. During this procedure, cardiopulmonary bypass (CPB) is used to take over the heart's function and keep blood circulating [3]. While CPB is a life-saving technology, it comes with its own risks, including inflammation, blood clotting issues, and challenges in maintaining stable blood pressure. These complications can slow recovery and affect long-term patient outcomes [4] [5] [6].

In this context, anaesthesia stabilises patients during surgery and improves recovery afterwards. One drug that has been getting a lot of attention in cardiac surgery is dexmedetomidine (DEX). DEX is a selective α2 adrenergic receptor agonist, which means it works by calming the nervous system and promoting relaxation without the heavy sedation seen with some other anaesthetics. It helps lower stress levels, provides pain relief, and has calming effects on the heart and blood vessels, which are particularly beneficial during heart surgery [7] [8] [9].

Patients undergoing valve replacement surgery, especially those with RHD, often face a range of challenges, like fluctuations in blood pressure, immune system activation, and even heart muscle injury. Research suggests that DEX can help by reducing the body's stress response, keeping the blood pressure stable, and even protecting the heart muscle from injury [10] [11] [12] [13]. In addition, DEX has been shown to influence the immune system by reducing the release of inflammatory markers - substances in the body that trigger inflammation after surgery. This could improve recovery times and reduce complications like infections and organ dysfunction [14].

One of the biggest issues during cardiac surgery is inflammation caused by CPB. This inflammation can lead to longer recovery periods and more severe complications. DEX appears to help manage this by regulating the immune response and promoting a balance that helps the body recover more efficiently. Studies have also found that DEX increases the ratio of CD4+ to CD8+ T-cells, which could indicate a more balanced and protective immune state, further supporting recovery after surgery. On top of that, DEX seems to reduce heart muscle injury, as measured by markers like troponins, which could lead to less damage and a smoother recovery.

This study aims to explore how DEX can be used in patients undergoing RHD valve replacement surgery. We'll focus on how DEX impacts hemodynamics, myocardial injury markers, and immune function during the procedure. By examining these key factors, we aim to understand whether DEX can help reduce postoperative inflammation, shorten recovery time, and ultimately improve the prognosis for these patients. In doing so, this study hopes to offer insights into how DEX can be used more effectively in cardiac surgery and help refine post-surgery treatment strategies.

## Materials and methods

### Subjects

A total of 140 patients who underwent CPB cardiac valve replacement under general anaesthesia at West China Hospital of Sichuan University from January 2022 to December 2024 were enrolled. The random number table method divided them into two groups, with 70 patients in each. There were 39 males and 31 females in the controls, aged from 55 to 67 years old, with 32 cases of American Society of Anesthesiologists (ASA) grade II and 38 cases of grade III, 27 cases of New York Heart Association (NYHA) heart function grade II and 43 cases of grade III. The observation group had 26 males and 44 females, 46~68 years old, ASA grade II in 35 cases, grade III in 35 cases, NYHA heart function grade II in 32 cases, and grade III in 38 cases. There was no obvious difference in the general information of subjects (*P*>0.05). The Medical Ethics Committee of West China Hospital of Sichuan University approved this trial.

### Inclusion and exclusion criteria

Inclusion criteria: The patient's indicators met the surgical indications and completed the operation. The patients had left ventricular ejection fraction (EF) 30% and no left atrial thrombus. This was the first time that the patient underwent heart valve replacement, and the replacement valve was a mechanical valve; ASA classification was II-IV and NYHA classification was II-III. All patients or their families volunteered to participate in this experiment and signed informed consent.

Exclusion criteria: Age <18 and >70 years old; Patients with severe dysfunction of liver, lung, kidney, and other organs; Allergy to the drug in this trial; Persons with communication disorders (language disorders, neurological history, psychiatric disorders) [13] [14].

### Methods

The patient took two tablets of diazepam (Shanghai Shangyao Sine Pharmaceutical Co., LTD., H31021151, 2.5 mg/tablet) for preoperative anxiety one day before surgery (at least 12 hours from the time of surgery). After the patients were admitted to the operating room, the venous access was opened, and routine electrocardiogram monitoring was performed. The vital signs such as heart rate (HR), blood pressure, mean arterial pressure (MAP), body temperature, and blood oxygen saturation were observed. After the indicators were confirmed to be normal, anaesthesia was induced. Midazolam injection (Jiangsu Jiuxu Pharmaceutical Co., LTD., H20153019, 3 mL:15 mg) 1 mg/kg, sufentanil citrate injection (Yichang Humanwell Pharmaceutical Co., LTD., H20054171, 50 μg/tablet) 2 μg/kg, etomidate emulsion injection (Jiangsu Nhwa Pharmaceutical Co., LTD., H20020511, 20 mg/tablet) 0.3 mg/kg, and vecuronium injection (Zhejiang Xianju Pharmaceutical Co., Ltd., H19991172, 4 mg/bottle) 0.015 mg/kg were injected successively. Tracheal intubation was performed.

After the eyelid reflex disappeared, the observation group was intravenously injected with DEX hydrochloride injection (Fuan Pharmaceutical Group Qingyutang Pharmaceutical Co., LTD., H20213633, C13H16N2 2 mL:0.2 mg) 0.5 μg/(kg*h) until the end of the operation. In the controls, the same volume of normal saline was injected intravenously at the same rate. Intraoperative anaesthesia was maintained using vein pump injection of propofol emulsion injection (Corden Pharma S.PA. Viale dell' Industria, 3, 20867 Caponago, Italy., H20171275, 500 mg/tablet) 4 mg/(kg*h) and hydrochloric acid fentanyl (Langfang branch, China National Pharmaceutical Industry Corporation Ltd., H20123421, Remifentanil C20H28N2O5 2 mg) 1 mg/(kg*h) to maintain the plasma concentration of 2-3 ng/mL. The vecuronium injection (0.015 mg/kg) was injected as needed to maintain muscle relaxation. CPB was performed with mild hypothermia at 30°C, perfusion flow was 2.0-2.4 L/(m^2^*min), and MAP was maintained at 50-80 mmHg, activated coagulation time at >480s, hematocrit at 25%-30%. The machine was stopped smoothly when the electrolyte and acid-base balance were stable. At the end of the operation, the patient was extubated when spontaneous breathing resumed.

### Observation indicators

### Perioperative indicators

The intraoperative recovery time of heartbeat, postoperative awakening time, postoperative spontaneous breathing recovery time, intensive care unit (ICU) residence time, and postoperative hospital stay time of subjects were compared.

The amount of intraoperative anaesthetics (midazolam, sufentanil, and propofol) was compared in the subjects.

### Hemodynamic indicators

Heart rate (HR) and mean arterial pressure (MAP) were calculated and compared before anaesthesia induction (T0), after anaesthesia induction (T1), ascending aortic cross-clamping (T2), cooling to 30°C (T3), rewarming to 37°C (T4), and termination of CPB (T5). The values of HR and MAP were measured after the indexes were stable.

### Myocardial injury indicators

Cardiac troponin I (cTnI) is a unique protein in myocardial cells. When myocardial cells are damaged, cTnI is released into the blood and remains in the blood for a long time. Creatine kinase-MB (CK-MB) is a special enzyme in cardiomyocytes released into the blood when cardiomyocytes are damaged. cTnI and CK-MB were used to measure myocardial injury [15].

3 mL venous blood was collected from the patient and placed into an anticoagulant tube containing ethylene glycol diamine tetraacetic acid (EDTA). The anticoagulant tube was slowly and gently inverted to allow the blood to mix well with the anticoagulant. The cTnI assay kit (Nanjing Norman Biological Technology, China) was used for detection. The samples were centrifuged in an Allegra 64R high-speed refrigerated centrifuge (Beckman Coulter, USA) at 3,000 r/min for 10 min, and the supernatant was taken. The samples were stored at -80°C until all the samples were collected. 20 μL of sample was thawed and added to 160 μL of potassium dihydrogen phosphate (12 g/L), dipotassium hydrogen phosphate (18 g/L) and polyethylene glycol 6000 (25 g/L) mixture and incubated at 37°C for 5 min. Antihuman cTnI latex particle suspension 40 μL (8 g/L) was added to the mix. Incubation was carried out at 37°C for 5 min. The absorbance A1 and A2 were recorded at 1 min and 5 min, respectively, and the difference A=A2-A1 was computed. The cTnI was calculated as described in Equation (1).


(1) \begin{array}{c} cTnl=\frac{\Delta A_{measure} -\Delta A_{blank} }{\Delta A_{calibration} -\Delta A_{blank}}\times C_{calibration}
\\ (1)1  \end{array}

The CK-MB assay kit (Guangzhou KOFA Biotechnology Co., Ltd., China) was adopted to detect the patient's level of CK-MB. 3 mL venous blood was centrifugated, the supernatant was removed, and the sample was tested adopting the Siemens ADVIA1650 automatic biochemical analyser. The CK-MB calculation method was presented in Equation (2).


(2) \begin{array}{c} CK-MB=\frac{\Delta A_{measure/min} -\Delta A_{blank/min} }{\Delta A_{calibration/min} -\Delta A_{blank/min}}\times C_{calibration}
\\ (2)2  \end{array}

The levels of cTnI and CK-MB at T0, T5, the end of the operation (T6), 8 hours following operation (T7), and 24 hours following operation (T8) were recorded and compared.

### Indexes of cellular immune function

CD4^+^ T cells are known as helper T cells, which regulate and coordinate the immune response, helping other immune cells in the immune response. In contrast, CD8^+^ T lymphocytes, known as cytotoxic T cells, can recognise and destroy pathogen-infected cells. The CD4^+^/CD8^+^ ratio is the ratio of CD4^+^ T cells to CD8^+^ T cells, reflecting the body's immune regulation and response balance. CD4^+^, CD8^+^ and CD4^+^/CD8^+^ ratios were used as indicators to measure cellular immune function [16].

6 mL peripheral venous blood was collected from the patients before and after surgery, and the levels of CD4^+^ and CD8^+^ were measured using a NovoCyte Advanteon B7 flow cytometer (Agilent, USA), and the CD4^+^/CD8^+^ ratio was computed.

### Indicators of inflammatory factors

Interleukin-8 (IL-8) is a chemokine that attracts and activates white blood cells to sites of inflammation. IL-10 is an anti-inflammatory cytokine that helps to limit excessive amplification of the inflammatory response and maintain a steady state of the immune system. Tumour necrosis factor-α (TNF-α) can activate immune cells and enhance inflammatory and immune responses [17] [18]. IL-8, IL-10, and TNF-α were used as the standard to measure the changes in inflammatory factors.

The IL-8, IL-10, and TNF-α levels were measured by enzyme-linked immunosorbent assay (ELISA). 3 mL venous blood was collected before, after, and 24 hours following the operation and then put into an Allegra 64R high-speed refrigerated centrifuge at 3,000 r/min for 10 min. The supernatant was taken. The sample was stored at -80°C until all samples were taken and tested. IL-8 ELISA kit (Xiamen Lunchangshuo Biotechnology Co., LTD., China), IL-10 ELISA kit (Shanghai Yaji Biotechnology Co., LTD., China), and TNF-α ELISA kit (Shanghai Jingkang Biological Engineering Co., LTD., China) were adopted to measure the levels of IL-8, IL-10, and TNF-α before and after surgery.

### Occurrence of adverse reactions

All adverse reactions, including vomiting, bradycardia, abnormal blood pressure, vertigo, respiratory depression, and somnolence, were recorded until discharge.

### Statistical analysis

SPSS 26.0 software was employed for data processing. Measurement data were expressed as mean ± standard deviation. (x̄ ± s), and a *t*-test was adopted. Count data were presented using frequency or rate, and χ^2^ was adopted. *P*<0.05 was considered statistically significant.

## Results

### Demographic and clinical characteristics of study participants

A total of 140 patients (70 in the observed group and 70 in the control group) were included in the study. The mean age of participants was 55.4±10.2 years, with a male-to-female ratio of 1:1.2. All patients had a diagnosis of rheumatic heart disease requiring valve replacement surgery. Demographic characteristics, including age, gender, and preoperative comorbidities, were similar between the two groups (P>0.05), ensuring the comparability of the groups.

### Comparison of perioperative indicators

**Figure 1:**
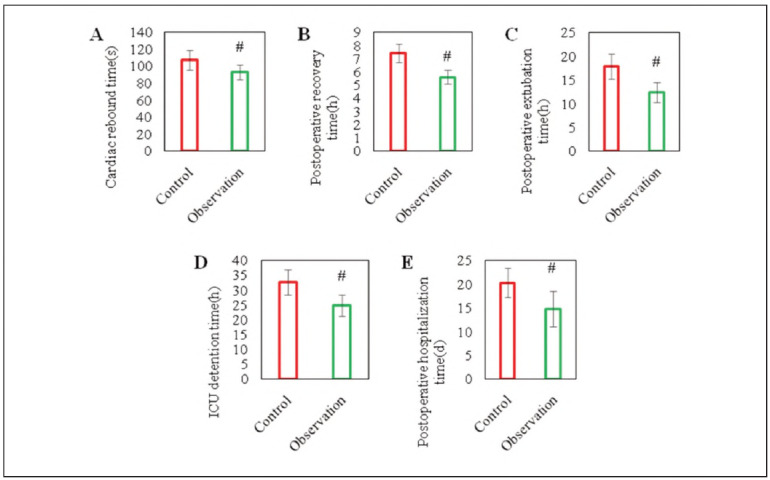
Comparison of important perioperative time indicators.<br>(A: time of recovery of heartbeat; B: postoperative recovery time; C: postoperative extubation time; D: length of ICU stay; E: length of hospital stay; Note: # as against controls, P<0.01)

The recovery time of heartbeat (93±9) s, postoperative awake time (5.6±0.5) h, postoperative extubation time (12.4±2.1) h, postoperative ICU stay time (24.7±3.7) h, hospital stay (14.8±3.7) d of observed subjects were visibly lower as against the controls (107±12) s, (7.4±0.7) h, (17.9±2.6) h, (32.6±4.2) h, and (20.3±3.1 ) d (*P*<0.01) (Figure 1). This means that DEX has a positive outcome on the recovery process of postoperative patients, and it has the effects of regulating the patient's cardiovascular system, sedation, and analgesia, improving the overall rehabilitation of patients.

**Figure 2:**
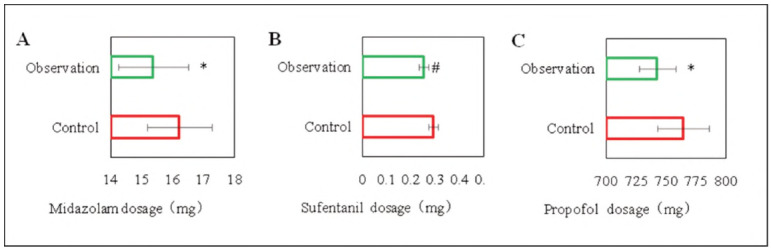
Comparison of perioperative anesthetic use.<br>(A: midazolam; B: sufentanil; C: propofol; Note: # relative to controls, P<0.01, * relative to controls, P<0.05)

Figure 2 illustrates the perioperative midazolam, sufentanil, and propofol usage in the subjects. The use of midazolam, sufentanil, and propofol in observed subjects was lower than controls (*P*<0.05). This meant that DEX could reduce anaesthetic use during RHD valve replacement.

### Hemodynamic parameters

**Figure 3:**
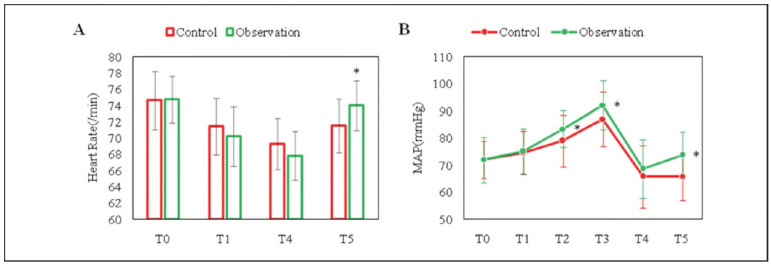
Contrast of hemodynamic parameters at various time points.<br>(T0: before induction of anesthesia, T1: after induction of anesthesia, T2: ascending aortic cross-clamping, T3: cooling to 30°C, T4: rewarming to 37°C, T5: termination of CPB; * As against controls, P<0.05)

CPB was performed at T2 and T3, so HR=0/min. At T0-T1, HR decreased with the progress of anaesthesia, but there was no visible distinction in the HR of subjects *(P*>0.05). At T4-T5, HR showed an upward trend. The HR of observed subjects at T5 (74.97±3.05)/min was visibly higher relative to controls (71.52±3.26)/min (*P*<0.05) (Figure 3A). The MAP of subjects presented a trend of first increasing and then decreasing with the surgical process. The MAP of controls reached its peak at T3, while the MAP of observed subjects reached its peak at T2, and then it began to decrease. The observed subjects' MAP was visibly higher than the controls at T2, T3, and T5 (*P*<0.05). In recovery, DEX can relieve the patient's stress response, helping stabilise the HR (Figure 3B).

### Contrast of myocardial injury indicators

**Figure 4:**
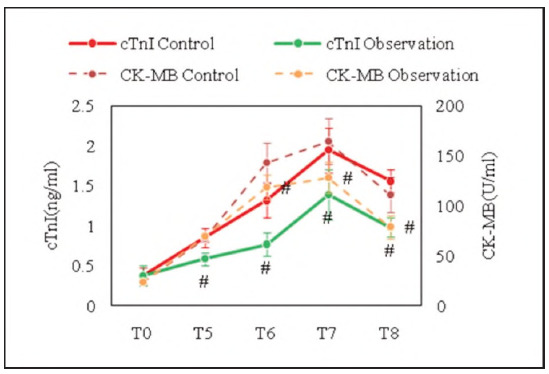
Contrast of myocardial injury indicators at different time points.<br>(T0: before induction of anesthesia, T5: termination of CPB, T6: end of surgery, T7: 8 hours after surgery, T8: 24 hours after surgery; # as against controls, P<0.01)

cTnl and CK-MB of subjects at different time points suggested a trend of first increasing and then decreasing over time after the end of surgery (Figure 4). The levels of cTnl in observed subjects at T5-T8 were markedly lower as against the controls (*P*<0.01), and the levels of CK-MB in observed subjects at T6-T8 were markedly lower as against the controls (*P*<0.01). The lower levels of cTnl and CK-MB in observed subjects indicate that DEX-treated patients perform better in myocardial damage; DEX has some protective outcomes on the myocardium.

### Contrast of cellular immune function indexes

**Figure 5:**
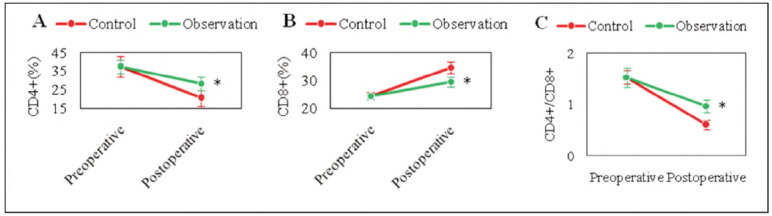
Contrast of cellular immune function.<br>(A: CD4^+^; B: CD8^+^; C: CD4^+^/CD8^+^; * as against controls, P < 0.05)

The cellular immune function of subjects before and after surgery suggested that, as against the controls, CD4^+^ and CD4^+^/CD8^+^ were markedly high, and CD8^+^ was considerably lower in the observed subjects (*P*<0.05). DEX can effectively improve the cellular immune function (Figure 5).

### Contrast of inflammatory factors

**Figure 6:**
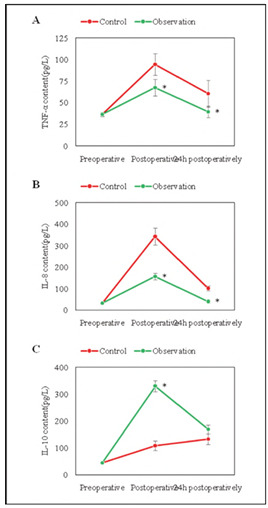
Contrast of inflammatory factors at various time points.<br>(A: TNF-α; B: IL-8; C: IL-10; * as against controls, P < 0.05)

The subjects' IL-8, IL-10, and TNF-α suggested a trend of increasing first and then decreasing, and there was no visible distinction before operation (*P*>0.05). IL-8 and TNF-α in observed subjects were markedly lower as against the controls immediately after surgery and 24 hours after surgery (*P*<0.05). The level of IL-10 in observed subjects was considerably higher than that of the controls immediately post-surgery. There was no visible distinction in IL-10 at 24 hours following operation (*P*>0.05), but it was markedly higher relative to before operation (*P*<0.05). The immune response induced by surgical trauma can lead to the high expression of inflammatory factors. These results indicate that DEX can promote the release of anti-inflammatory factors and reduce the inflammatory response (Figure 6).

### Contrast of adverse reactions

**Figure 7:**
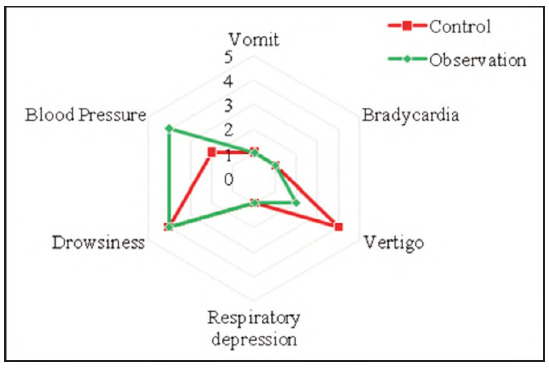
Contrast of adverse reactions in subjects (person).

Figure 7 illustrates the incidence of postoperative adverse reactions. In the controls, there were 1 case of vomiting (1.43%), 1 case of bradycardia (1.43%), 2 cases of abnormal blood pressure (2.86%), 4 cases of vertigo (5.71%), 1 case of respiratory depression (1.43%), and 4 cases of drowsy (5.71%). The total incidence of adverse reactions was 18.57% (13/70). Observed subjects had 1 case of vomiting (1.43%), 1 case of bradycardia (1.43%), 4 cases of abnormal blood pressure (5.71%), 2 cases of vertigo (2.86%), 1 case of respiratory depression (1.43%), and 3 cases of drowsiness (4.29%). The total incidence of adverse reactions was 17.14% (12/70). There was no visible distinction in the incidence of adverse reactions between subjects and different adverse reactions (*P*>0.05). The results indicate that DEX treatment can effectively control intraoperative analgesia and inflammatory postoperative response and has a similar effect with traditional treatment (controls) in controlling postoperative adverse reactions.

## Discussion

Heart valve replacement under CPB is a surgical method used to treat severe heart valve disease, during which the heart of sick persons will be stopped, and their blood circulation function will be replaced by CPB equipment [19]. In addition to the complications of conventional cardiac surgery, CPB itself may cause a series of complications, such as abnormal coagulation function, inflammatory response, and lung injury in sick persons. Therefore, choosing a safe and effective anaesthesia program is particularly important.

DEX is a commonly used anaesthetic drug in clinical practice, which is often used for analgesia, ICU management, and neurological monitoring and examination [20]. This article experimented with the use of DEX as an adjunct to conventional anaesthesia after anaesthesia induction in sick persons. The perioperative recovery time of observed subjects assisted by DEX was found to be shortened while the anaesthetic dose was markedly reduced. This not only helps to avoid the risk of anaesthesia-related complications but also alleviates the stress response during surgery and helps to maintain hemodynamic stability in sick persons. DEX reduces sympathetic impulses through central and peripheral activation of α2 adrenergic receptors, which reduces vasodilatation and blood pressure [21] [22]. During procedures such as ascending aortic occlusion, sick persons experience a certain state of vascular tone, while observed subjects' blood vessels are modulated by DEX, making the tendency for vasodilation and blood pressure to rise more pronounced. Tan et al. [23] found that 0.5 μg/kg*h DEX can change the permeability of ion channels, effectively maintain perioperative cardiac electrophysiological stability, prolong the effective refractory period of cardiomyocytes, and reduce myocardial autonomy. This result is consistent with previous studies and confirms the effectiveness of DEX as a sedative.

DEX has been found to play a positive role in postoperative myocardial protection. DEX alleviates postoperative inflammatory response by regulating immune and inflammatory factors, thereby reducing the degree of myocardial injury. This article showed that in observed subjects after surgery, the levels of CD4^+^ T cells increased markedly, while the levels of CD8^+^ T cells decreased, resulting in an increased CD4^+^/CD8^+^ ratio, implying that the immune system of observed subjects was more likely to maintain an appropriate immune response. It indicates that the body is more likely to be in a state of immune tolerance [24] [25]. This state helps fight off infections, reduce the risk of autoimmune diseases, and better cope with illness and trauma. Chen et al. [26] found that in the adaptive immune response, DEX had little effect on the humoral response of B cells but could enhance cellular immunity by regulating the differentiation, number, and proportion of T cell subtypes.

In controlling inflammation factor levels, DEX can effectively reduce the IL-8 and TNF-α levels and improve the IL-10, IL-8, and TNF-α as inflammatory factors, suggesting that DEX can reduce postoperative inflammatory reactions and exert anti-inflammatory outcomes [27]. IL-10 can inhibit the production of other inflammatory factors, such as TNF-α and interferon-γ, thereby reducing excessive inflammatory response. Therefore, high expression of IL-10 helps inhibit inflammatory response and maintain the immune system's balance [28] [29]. Dong et al. [30] found that DEX could improve cognitive dysfunction in rats undergoing CPB by reducing the inflammatory response around the heart. This article compared the adverse reactions of the two groups, and no statistically visible distinction was found. This indicates that intravenous DEX infusion during surgery does not lead to increased adverse reactions in sick persons; that is, it is highly safe [31] [32].

Chen et al. [10] investigated the effects of dex- medetomidine (DEX) on myocardial injury and inflammation during rheumatic heart valve replacement surgery, finding that continuous DEX infusion reduced myocardial injury markers (cTnI), inflammation (IL-8), and oxidative stress, with improved cardiac function. Similarly, our study demonstrated that DEX significantly lowered myocardial injury markers (cTnI and CK-MB) and inflammatory markers (IL-8, TNF-α), increasing IL-10 levels in patients undergoing valve replacement for rheumatic heart disease. Additionally, we found that DEX supported hemodynamic stability and improved immune function, with higher CD4^+^ and CD4^+^/CD8^+^ ratios postoperatively. Both studies highlight DEX's role in reducing myocardial injury and inflammation, though our study further suggests its potential to modulate immune responses, offering new insights into its broader therapeutic effects.

Shan et al. [33] explored the cardioprotective effects of sevoflurane during double valve replacement surgery, finding that it reduced myocardial injury markers (CK-MB, cTnI) and inflammatory cytokines (IL-6, IL-8, TNF-α) while increasing levels of heat shock protein-70 (HSP70) and superoxide dis- mutase (SOD) activity. Similarly, our study examined the effects of dexmedetomidine (DEX) on myocardial injury and inflammation during rheumatic heart disease valve replacement surgery. Both studies highlighted the beneficial effects of anaesthetic agents on reducing myocardial injury and inflammation, though the mechanisms differed: sevoflurane's cardioprotective effect was linked to HSP70 upregulation, while DEX modulated immune responses, increasing IL-10 and improving the CD4+/CD8+ ratio. Our findings support DEX's role in reducing myocardial injury markers and inflammatory responses, complementing the cardioprotective effects of sevoflurane, and suggest that DEX may have additional benefits in modulating immune function.

Netala et al. [34] provided a comprehensive review on the management of cardiovascular diseases (CVDs), emphasising the role of cardiac biomarkers such as cTnI, CK-MB, IL-6, and TNF-α in diagnosing and monitoring CVD progression. Their review also highlighted various imaging modalities and pharmacotherapies for managing CVDs, including antiinflammatory treatments. Similar to their findings, our study focused on the impact of dexmedetomidine (DEX) on myocardial injury markers, including cTnI and CK-MB, during rheumatic heart disease valve replacement surgery. Both studies underline the importance of cardiac biomarkers in evaluating treatment efficacy. While Netala et al. [34] discuss a broad spectrum of treatments for CVD, including pharmacological and herbal interventions, our research specifically explores the potential of DEX to reduce myocardial injury and inflammation. This suggests that DEX could be integrated as a targeted approach in the broader spectrum of cardiovascular management, particularly in surgical settings, complementing other therapeutic strategies.

A key limitation of this study is the short followup period, restricting the assessment of the long-term effects of dexmedetomidine on recovery and complications. The single-centre design and lack of blinding in treatment administration could also introduce bias and limit the generalizability of the findings. Additionally, the sample size, while sufficient, may not fully represent diverse patient populations with varying comorbidities.

## Conclusion

This study highlights the potential benefits of dexmedetomidine (DEX) in patients undergoing heart valve replacement under cardiopulmonary bypass (CPB). DEX reduced the need for anaesthetic drugs, maintained hemodynamic stability, and protected myocardial function by modulating inflammatory responses. It improved immune balance by increasing the CD4+/CD8+ ratio and reducing inflammatory markers like IL-10 and TNF-α. These findings suggest that DEX can enhance perioperative outcomes and reduce complications in valve replacement surgeries, particularly for patients with rheumatic heart disease. Further research is needed to optimise dosing and better understand its mechanisms in this context.

## Dodatak

### Conflict of interest statement

All the authors declare that they have no conflict of interest in this work.
